# Chronic myeloid leukaemia: have basophils gone the way of the Dodo bird?

**DOI:** 10.1038/s41375-026-02910-9

**Published:** 2026-03-16

**Authors:** Lucia Turcsanyi, Xiao-Shuai Zhang, Milos Kudelka, Kristyna Kubikova, Martin Radvansky, Sen Yang, Eva Kriegova, Jarmila Juranova, Jiri Mayer, Hana Klamova, Petra Belohlavkova, Michal Karas, Lukas Stejskal, Eduard Cmunt, Megan Othus, Robert Peter Gale, Qian Jiang, Edgar Faber

**Affiliations:** 1 Department of Hemato-Oncology, Olomouc University Hospital and Faculty of Medicine and Dentistry, Palacký University, Olomouc, Czech Republic; 2 Peking University People’s Hospital, Peking University Institute of Hematology, National Clinical Research Center for Hematologic Disease, Beijing, China; 3 Department of Computer Science, Faculty of Electrical Engineering and Computer Science, VSB-Technical University of Ostrava, Ostrava, Czech Republic; 4 Department of Immunology, Olomouc University Hospital and Faculty of Medicine and Dentistry, Palacký University, Olomouc, Czech Republic; 5 Department of Internal Medicine Hematology and Oncology, University Hospital Brno and Masaryk University, Brno, Czech Republic; 6 Institute of Hematology and Blood Transfusion, Prague, Czech Republic; 7 4th Department of Internal Medicine and Hematology, University Hospital Hradec Kralove and Charles University, Hradec Kralove, Czech Republic; 8 Department of Hemato-oncology, University Hospital Pilsen and Charles University, Pilsen, Czech Republic; 9 Department of Hematooncology, University Hospital Ostrava, Ostrava, Czech Republic; 10 1st. Internal Department, General University Hospital Prague, Prague, Czech Republic; 11 Fred Hutchinson Cancer Research Center, Seattle, WA USA; 12 Centre for Haematology, Department of Immunology and Inflammation, Imperial College London, London, UK; 13 Peking University People’s Hospital, Qingdao, China

**Keywords:** Risk factors, Chronic myeloid leukaemia

For over 40 years, percentage blood basophils has been used to estimate survival and to define the accelerated phase [1–6]. Recently, this use has declined or disappeared, especially since the introduction of tyrosine kinase inhibitor (TKI) therapy. No widely used CML survival prediction model includes percentage blood basophils [7]. Putting a nail in the coffin, the 2022 WHO classification of myeloid neoplasms, for good reason, eliminated accelerated phase CML making percentage blood basophils irrelevant [8]. In contrast, the 2022 International Consensus Classification (ICC) retained accelerated phase CML. The 2025 European LeukemiaNet classification seems undecided [9, 10] (reviewed in refs. [11, 12]).

It’s interesting to consider how and why percentage blood basophils came into prominence in CML, and why percentage and not concentration was the unit of quantification, like haemoglobin (g/L) and WBCs (*x*10^9^/L). An explanation is that automated particle counters able to estimate basophil concentration became widely used beginning in the late 1980s. Previously, basophil quantity was assessed only by a visual blood cell differential estimate resulting in percentage. But now, if percentage blood basophils has gone the way of the Dodo bird, last sighted in 1662, might there be residual value were blood basophils to be quantified as a concentration?

The units of how a quantity is expressed are quirky. Consider a *shrewdnes*s of apes, a *congregation* of alligators, a *murder* of crows, a *mob* of kangaroos, a *parliament* of owls, *etc*. So it is reasonable to ask: Why was the quantity of blood basophils previously expressed as a percentage, and is this quantification important??

Today, four methods are widely used to quantify blood basophils: (1) visual differential analyses; (2) automated particle counting; (3) multi-parameter flow cytometry (MPFC); and (4) artificial intelligence (AI) and machine learning.

Visual differential analyses of WBCs are the reference standard for basophil enumeration despite well-known limitations including non-uniform distribution, observer variability and statistical limitations [13]. In acute leukaemias, visual differential analyses of 100 WBCs are recommended by the International Council for Standardization in Haematology [14]. There are no corresponding recommendations for CML. Articles by Sokal et al., Baccarani et al., Pfirrmann et al. and Apperley et al. and others do not specify how to determine percentage blood basophils [6, 7, 9, 15].

Consider the statistical limitations of a visual differential analysis of 100 WBCs. Assume a basophil can be precisely visually identified (questionable at best). Next, assume an observer identifies 5 basophils amongst 100 WBCs (5%). This value has a 95% Confidence Interval of 0.7, 9% meaning were the same procedure repeated 99 more times, 95% of the values would be within this range. (Another 4% would be lower or higher). It is a common misconception that the *true value* lies within the 95% Confidence Interval. However, this huge uncertainty over the true percentage blood basophils would be even greater if the same observer looked a different section of the microscope slide, if a different observer looked at the same section of the microscope slide, if different observers looked at different slides and so on. Because of this imprecision someone could be classified as having a poor prognosis or not, or being in accelerated phase or not. Misclassification can have therapy implications, some of which could be lethal. (*The slip of a lip* [*Can sink a ship*]; *Duke Ellington, 1942*). Despite the obvious limitations, a visual WBC differential analysis remains the *gold standard* against which the accuracy of other methods is judged.

Is quantifying blood basophil percentage using an automated particle counter, like a Coulter^®^ counter, which samples tens of thousands of WBCs, more accurate? No. 1st, particle counters do not directly quantify percentage but rather concentration. However, the accuracy of automated particle counters for blood basophil concentration is generally poor, with some giving erratic or unreliable results, such as failing to detect increased blood basophil concentrations in people with CML. Automated counters are precise for other cell types but not for basophils, resulting in underestimation compared with visual differential or MPFC quantification [16]. Most haematologists probably do not realize percentage basophils on a report from an automated particle counter is obtained by dividing the often-inaccurate basophil concentration by the WBC. For these reasons, the US National Institutes of Health (US NIH) recommends against using automated particle counter data to quantity blood basophil concentration or percentage [17].

Most recently, MPFC has been used to quantify blood basophils using monoclonal antibodies to CD123, CD193, HLA DR and FcɛRI [18]. The challenge is what to consider the *gold standard*. Visual quantification and MPFC are more accurate compared with automated particle counting in identification of atypical basophils [19]. The bottom line is that the 3 methods quantifying blood basophil percentage or concentration are inaccurate, but visual enumeration remains the reference standard. In the future, artificial intelligence (AI) and machine learning models may improve accuracy, but we are left with the dilemma: accurate compared to what? Machine learning requires a gold standard to be educated by. Presently, we have only an arbitrary gold standard, the visual differential analysis of WBCs with the limitations discussed above. An automated microscope could scan 1000 s instead of 100 of WBCs, useful if we were certain which cell is a basophil.

A 4th approach to quantifying diverse blood cells, including basophils, uses AI, including digital image processing and/or machine learning, like the Regional Convolutional Neural Network (R-CNN) and You Only Look Once (YOLO) methods [20, 21]. Specific machine learning models for blood basophils are also available [22]. These techniques are not yet standardized but are likely to become widespread.

When prognostic co-variates for CML were 1st identified, numbers of basophils estimated by automatic particle counters and MPFC were not widely available, whereas percentage basophils were easily obtainable from a visual analysis of 100 WBCs [1–4, 7]. This may explain the reliance on basophil percentage rather than concentration persisted despite advances in blood cell quantification. Another consideration is temporality. Use of basophil percentage as a prognostic covariate was predominantly before TKI-therapy replaced other therapies of CML. Some predictive scores during the TKI era, such as EURO and EUTOS score retained basophil percentage [3, 4]. However, basophil concentration was not evaluated in these studies.

Given this background, we sought to determine whether blood basophil concentration or percentage is a better outcome predictor in CML. To do so, we first interrogated a single-centre dataset of 131 subjects with newly diagnosed chronic phase CML receiving TKI-therapy using patient similarity networks (PSNs) to determine if blood basophil percentage or concentration was a more accurate predictor of CML-related failure. We found basophil concentration was more accurate (Fig. 1). We validated our finding in a dataset of 1870 Chinese subjects with CML in chronic phase also receiving TKI-therapy (Fig. 2). Lastly, we interrogated data from 1409 subjects with chronic phase CML receiving TKIs 2005–2020 in the Czech Republic (Fig. 3A–E). In multi-variable Cox regression models neither blood basophil percentage nor concentration was predictive of any outcome including failure-free survival (FFS), progression-free survival (PFS), CML-specific survival and event-free survival (EFS; Fig. 3B–E).

**Fig. 1:**
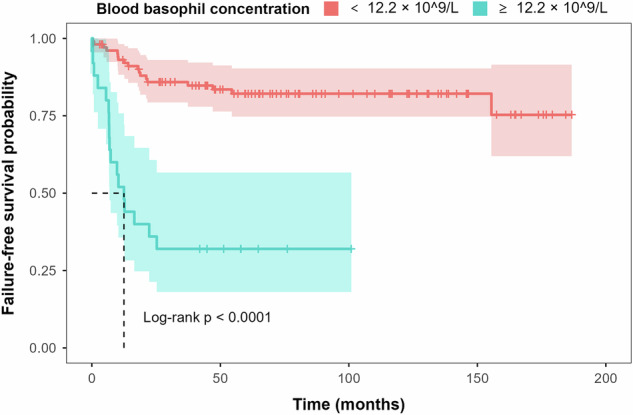
Failure-free survival by blood basophil concentration (≥12.2 ×10^9^/L) in 131 subjects with chronic phase chronic myeloid leukaemia treated with tyrosine kinase inhibitors at single centre. Failure-free survival estimated by Kaplan-Meier method compared patients according their blood basophil concentration at diagnosis (≥12.2 x 10^9/L and \lt 12.2 x 10^9/L). Failure-free survival was defined as the interval from TKI-start until haematologic progression, progression to blast phase or CML-related death therapy-failure defined as >10% BCR::ABL1 transcripts at 6 months and >1% BCR::ABL1 transcripts after 1 year, a TKI-resistant ABL1 mutation or high-risk additional cytogenetic abnormalities.

**Fig. 2:**
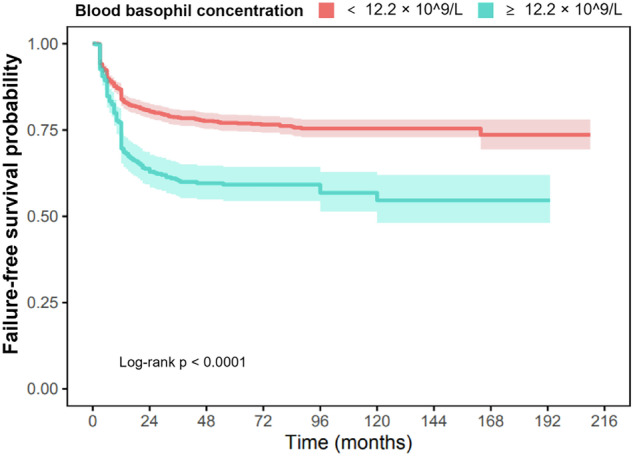
Failure-free survival in 1870 subjects with chronic phase chronic myeloid leukaemia receiving tyrosine kinase inhibitors by blood basophil concentration ≥12.2 ×10^9^/L. Failure-free survival estimated by Kaplan–Meier method compared patients according their blood basophil concentration at diagnosis (≥12.2 x 10^9/L and \lt 12.2 x 10^9/L). Failure-free survival was defined as the interval from TKI-start until haematologic progression, progression to blast phase or CML-related death therapy-failure defined as >10% BCR::ABL1 transcripts at 6 months and >1% BCR::ABL1 transcripts after 1 year, a TKI-resistant ABL1 mutation or high-risk additional cytogenetic abnormalities.

**Fig. 3:**
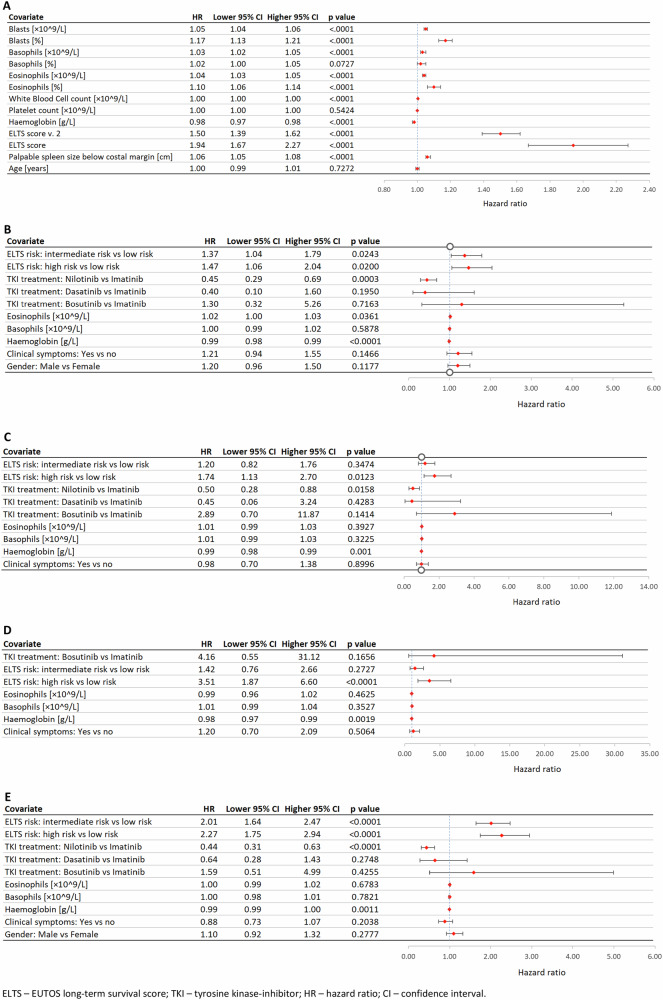
Forest plot of co-variate effects on risk of failure within uni-variate Cox models in 1409 subjects with chronic phase chronic myeloid leukaemia receiving tyrosine kinase inhibitors. In ELTS score v. 2 blood blast concentration was used instead of percentage for score estimation (**A**). Forest plots of adjusted covariate effects within multivariate Cox regression model for risk of failure (**B**), risk of progression (**C**), risk of CML-related death (**D**) and risk of event (**E**) in 1409 subjects with chronic phase chronic myeloid leukaemia receiving tyrosine kinase inhibitors. Presence of clinical symptoms was assessed at diagnosis. Percentage and concentration of blasts, basophils and eosinophils were estimated in blood.

The values of blood basophil concentration derived directly from automated blood particle counters were not collected in CML subject datasets, so we obtained these values as the product of basophil percentage by visual counting of 100 WBCs and WBC concentration. As such, these covariates are confounded. But which one(s) should we use to estimate CML-related failure, and does it really make a difference? Results of our studies in the two large datasets are contradictory and do not allow us to draw a conclusion.

Why the fuss over basophils now that no prognostic model uses basophil percentage or concentration to estimate survival or define accelerated phase has gone the way of the Dodo bird. Is it as Gertrude Stein said: *A rose is a rose is a rose is a rose*? We think not. Accuracy in science is important. It is likely that technical advances, AI and machine learning will improve the accuracy of visual WBC differential analyses.

In conclusion, we found no reason why percentage blood basophils rather than concentration was widely used in the context of CML. However, using concentration (derived from WBCs and visual counting) instead of percentage does not improve prediction accuracy by a substantial margin. An attempt to reintroduce basophils as the prognostic marker in CML should be repeated when improved quantitation of basophils is developed using AI-assisted digital processing and machine learning methods like R-CNN and YOLO.

## Data Availability

Available on reasonable request to EF or QJ consistent with the laws of the Czech Republic and Peoples Republic of China.
